# Lowering EphA4 Does Not Ameliorate Disease in a Mouse Model for Severe Spinal Muscular Atrophy

**DOI:** 10.3389/fnins.2019.01233

**Published:** 2019-11-19

**Authors:** Lindsay Poppe, Silke Smolders, Laura Rué, Mieke Timmers, Annette Lenaerts, Annet Storm, Lies Schoonaert, Antina de Boer, Philip Van Damme, Ludo Van Den Bosch, Wim Robberecht, Robin Lemmens

**Affiliations:** ^1^Department of Neurosciences, Experimental Neurology and Leuven Brain Institute (LBI), KU Leuven – University of Leuven, Leuven, Belgium; ^2^Laboratory of Neurobiology, VIB – KU Leuven Center for Brain & Disease Research, Leuven, Belgium; ^3^Department of Neurology, University Hospitals Leuven, Leuven, Belgium

**Keywords:** spinal muscular atrophy, EphA4, neuromuscular junction, sprouting, motor neuron

## Abstract

EphA4 is a receptor of the Eph-ephrin system, which plays an important role in axon guidance during development. Previously, we identified EphA4 as a genetic modifier of amyotrophic lateral sclerosis (ALS) in both zebrafish and rodent models, via modulation of the intrinsic vulnerability, and re-sprouting capacity of motor neurons. Moreover, loss of EphA4 rescued the motor axon phenotype in a zebrafish model of spinal muscular atrophy (SMA). Similar to ALS, SMA is a neurodegenerative disorder affecting spinal motor neurons resulting in neuromuscular junction (NMJ) denervation, muscle atrophy and paralysis. In this study, we investigated the disease modifying potential of reduced EphA4 protein levels in the SMNΔ7 mouse model for severe SMA. Reduction of EphA4 did not improve motor function, survival, motor neuron survival or NMJ innervation. Our data suggest that either lowering EphA4 has limited therapeutic potential in SMA or that the clinical severity hampers the potential beneficial role of EphA4 reduction in this mouse model for SMA.

## Introduction

Spinal muscular atrophy (SMA) is an autosomal recessive neurodegenerative disorder, affecting spinal motor neurons within the central nervous system. Patients present with muscle weakness and paralysis resulting from neuromuscular junction (NMJ) dysfunction and denervation (Lunn and Wang, 2008; Van der Pol et al., 2012; Martínez-Hernández et al., 2013). SMA is the leading genetic cause of infant lethality, although there is a lot of variety in the clinical presentation with non-lethal adult forms at the other side of the spectrum (Lunn and Wang, 2008). This devastating disorder is caused by reduced levels of survival of motor neuron (SMN) protein, which is ubiquitously expressed and involved in many neuronal pathways, including RNA metabolism, actin cytoskeleton dynamics, axonal RNA transport, and synaptic vesicle release (Singh et al., 2017). Reduced levels of SMN are elicited by deletions and/or loss-of-function mutations in the *SMN1* gene (Lefebvre et al., 1995). Humans have one or several copies of a duplicated gene, *SMN2*. However, inefficient splicing due to a single nucleotide substitution in exon 7 of this gene predominantly causes the formation of an unstable, truncated SMN protein lacking exon 7 (SMNΔ7). Therefore, full-length SMN protein is only produced at 10% of the levels encoded by *SMN1* (Lorson et al., 1999; Monani et al., 1999). As SMA patients rely on *SMN2* for production of SMN protein, the number of *SMN2* gene copies determines the residual SMN protein levels and the onset and severity of the disease (Feldkötter et al., 2002). Nusinersen and Zolgensma are approved drugs for treatment of pediatric and adult SMA patients, and both increase the production of functional SMN protein. Whereas Nusinersen is an alternative splicing modulator of the *SMN2* gene, Zolgensma is a SMN1 gene replacement therapy (Hua et al., 2010; Finkel et al., 2017; Mendell et al., 2017). Still, other neuroprotective therapies could provide additional support for patients, or would be of importance for patients that are intolerant, not responsive to or excluded from SMN-targeting therapies (Talbot and Tizzano, 2017).

EphA4 is a tyrosine kinase receptor of the Eph-ephrin system which is highly expressed in the nervous system (Murai et al., 2003b). During development of the nervous system, EphA4 has an important role in axon guidance (Shi et al., 2007). In adults, hippocampal EphA4 is a crucial mediator of synapse morphology, functionality, and plasticity (Murai et al., 2003a; Fu et al., 2007; Shi et al., 2007). Although EphA4 expression levels decrease in adult life, EphA4 is considered as a major contributor in neurological disorders such as spinal cord injury, stroke, and Alzheimer’s disease (Goldshmit et al., 2011; Lemmens et al., 2013; Munro et al., 2013; Fu et al., 2014; Vargas et al., 2014). Previously we identified EphA4 as a modifier of amyotrophic lateral sclerosis (ALS) in both zebrafish and rodent models (Van Hoecke et al., 2012). Inhibition of EphA4 signaling slowed down disease onset and/or progression, and improved motor function in rodent models for ALS by reducing the vulnerability of motor neurons and enhancing NMJ innervation (Van Hoecke et al., 2012). The latter is probably caused by the increased sprouting and re-innervation capacity of motor axons upon EphA4 reduction as was shown in a model of sciatic nerve axotomy (Van Hoecke et al., 2012).

Interestingly, knockdown of EphA4 also rescued the axonal deficits in a zebrafish model for SMA (Van Hoecke et al., 2012), suggesting that the neuroprotective effect of EphA4 inhibition could translate to other motor neuron diseases. In this study, we aimed to further investigate the modifying potential of reducing EphA4 in NMJ innervation, motor neuron survival, motor function, and survival in a mouse model for SMA.

## Materials and Methods

### Mice

Mice were housed in the KU Leuven animal facilities with a 12 h light-dark cycle at a temperature of 20°C. Animals were given free access to standard rodent chow and water. All animal experiments were carried out in accordance with the National Institutes of Health guide for the care and use of Laboratory animals (NIH publications No. 8023, revised 1978). Experiments were designed to minimize animal discomfort and were approved by the Ethical Committee for Animal Research of the University of Leuven, Belgium (P097/2013 and P003/2019).

We used a previously generated and widely used transgenic mouse model for severe SMA (Le et al., 2005), further on referred to as the SMNΔ7 mouse model. Frozen sperm of SMNΔ7 mice (FVB.Cg-*Grm7^*Tg(SMN*2)89*Ahmb*^ Smn1^TM 1*Msd*^* Tg (SMN2^∗^delta7) 4299Ahmb/J; stock number: 005025) was kindly provided for revitalization by Dr. Achsel (University of Lausanne, Switzerland). Mice have only one *smn* gene of which removal is embryonically lethal (DiDonato et al., 1997; Schrank et al., 1997; Viollet et al., 1997). Therefore, in addition to homozygous deletion of the murine *smn* gene caused by insertion of a β-galactosidase cassette, SMNΔ7 mice carry homozygous transgenes for the human SMN2 (hSMN2) gene, and cDNA of SMN1 lacking exon7 (hSMNΔ7) to extend the lifespan to approximately 2 weeks (Le et al., 2005). Control (Ctrl) mice were wild-type (smn^+/+^) or heterozygous (smn^+/–^) for the murine smn allele and homozygous for hSMN2 and hSMNΔ7.

To delete EphA4 in SMNΔ7 mice, we crossbred these mice with EphA4^–/–^ mice (Dottori et al., 1998), which were kindly provided by Dr. Turnley in a C57/Bl6J background (University of Melbourne, VIC, Australia). EphA4^–/–^ mice were backcrossed to FVB/N background for more than 10 generations, before being intercrossed with SMNΔ7 mice, to obtain experimental mice in pure FVB/N background. We deleted EphA4 heterozygously in SMNΔ7 mice, as removal of one EphA4 allele (EphA4^+/–^) was sufficient to improve the disease phenotype in an ALS mouse model (Van Hoecke et al., 2012). Moreover, EphA4^–/–^ mice develop a “hopping gait” phenotype, and show low birth rates and reduced body weight during the first postnatal weeks (Dottori et al., 1998; Kullander et al., 2003; Van Hoecke et al., 2012). The following experimental groups were obtained: Ctrl-EphA4^+/+^ (smn^+/+^-EphA4^+/+^ and smn^+/–^ -EphA4^+/+^), Ctrl-EphA4^+/–^ (smn^+/+^-EphA4^+/–^ and smn^+/–^-EphA4^+/–^), SMA-EphA4^+/+^ (smn^–/–^-EphA4^+/+^) and SMA-EphA4^+/–^ (smn^–/–^-EphA4^+/–^). The day of birth was defined as postnatal day 0 (PND0). Both male and female mice were included, and due to the frequent birth of only one SMA pup per litter, it was not feasible to use littermate controls in each litter. All experiments were conducted by a researcher blinded for the genotype.

### Genotyping

Mice were genotyped using tail biopsies obtained at PND1. Both smn and hSMN2 genotypes were determined via regular PCR using the following primer pairs (Table 1): primer pair 1 to amplify a region in the murine smn gene, primer pair 2 to amplify a region of the β-galactosidase cassette, primer pair 3 to amplify the inserted SMN2 gene and primer pair 4 to amplify the non-inserted region. The hSMNΔ7 transgene was genotyped using qPCR using primer pair 5 together with a 5′-CTTCTGGACCACCAATAATTCCCCCACC-3′ probe. Digital droplet PCR (ddPCR) was used to genotype for EphA4 using a commercially available Taqman copy number assay (Mm00530479_cn, Thermo Fisher Scientific). A commercially available copy number assay targeting AP3B1 was used as a reference gene for both qPCR and ddPCR (10031245, Bio-Rad).

**TABLE 1:** Primer pairs used for genotyping of SMNΔ7 mice.

**Primer pairs**	**Primer 1**	**Primer 2**
1	5′-GTGTCTGGGCTGTAGG CATTG-3′	5′-GGCTGTGCCTTTTG GCTTATCTG-3′
2	5′-GCCTGCGATGTCGGTTT CCGCGAGG-3′	5′-CCAGCGCGGAT CGGTCAGACG-3′
3	5′-CTGACCTACCAGGGA TGAGG-3′	5′-GGTCTGTTCTACA GCCACAGC-3′
4	5′-CTGACCTACCAGGGA TGAGG-3′	5′-CCCAGG TGGTTTAT AGACTCAGA-3′
5	5′-TGCTGGCTGCCTC CATTT-3′	5′-GCATCATCAAGAGAATC TGGACAT-3′

### Phenotypic Assessment

Weight was measured from PND1 on. From PND2 onward, motor function was assessed using the righting reflex test every other day and the hind-limb suspension (HLS) test daily. During the righting reflex test, pups were placed on a flat surface on their backs, and the time to flip back to an upright position with all paws touching the bench was measured (with a cutoff of 60 s). The average of three consecutive trials with a 5-min resting period in between was calculated. The HLS test was performed using a 50 ml conical tube filled with cotton wool at the bottom and positioned upright in a tube holder. Pups were placed inside the tube with the hind paws over the rim of the tube and facing down. The time spent hanging before falling down in the tube, the number of pulls (attempts to get out of the tube using the hind-limb muscles), and the hind-limb score (a score based on the position of the hind-limbs and tail of the animal) were measured as previously described (El-Khodor et al., 2008). In addition, a quantitative HLS test score (HLST score) was calculated via insertion of previous parameters in the following equation: $H\,L\,S\,T=\left[\left(t\,i\,m\,e\,s\,p\,e\,n\,t\,h\,a\,n\,g\,i\,n\,g\right)+10\,\left(\#\,o\,f\,p\,u\,l\,l\,s\right)\right]\,x\,\frac{\left(H\,L\,S+1\right)}{4}$, as described by Heier and DiDonato (2009). For all parameters, averages of two consecutive sessions with a 5-min resting period were calculated. In control mice, both tests were conducted only until PND8, as they were already strong enough to right immediately and escape the tube. For survival analysis, mice were monitored daily until found dead.

### Immunoblot

To determine SMN and EphA4 protein levels, pups were euthanized with an overdose of Dolethal (20 mg/ml) on PND8. Whole spinal cords were collected, snap frozen in liquid nitrogen and stored at −80°C. Samples were homogenized in RIPA buffer (Sigma-Aldrich, R0278) with protease (cOmplete; Roche, 11697498001) and phosphatase (phosSTOP; Roche, 4906845001) inhibitors using the MagNaLyser oscillator (Roche). Protein concentration was determined with the Pierce BCA protein assay kit (Thermo Fisher Scientific, 23225). For electrophoresis, we used 4–20% precast acrylamide gels (Mini-PROTEAN^®^ TGX^TM^; Bio-rad, cat#456-1096) and 20 μg of protein were loaded for each sample. Proteins were transferred to Immobilon-P (PVDF) membrane (Millipore, IPVH00010) and subsequently blocked with 5% non-fat-dry milk (Blotting-Grade Blocker; Bio-Rad, cat#170-6404) and 5% bovine serum albumin (BSA; Serva Electrophoresis GmbH, 1193003) in Tris–buffered saline with 0.001% Tween^®^ (TBS-T) for 1 h at room temperature (RT). Membranes were incubated with the following primary antibodies in TBS-T with 1% BSA: C-terminal mouse anti-EphA4 (1/500; Invitrogen, 37-1600), mouse anti-SMN Clone 8 (1/5000; BD Biosciences, 610646), and mouse anti-β-actin (1/5 000; Sigma, A54411). An anti-mouse-HRP antibody (1/5 000, DAKO) was used as secondary antibody and was diluted in TBS-T containing 5% non-fat-dry milk. ECL or FEMTO ECL (Thermo Fisher Scientific, 32106 and 34095) was used as a substrate and the signal was detected using LAS4000 (GE Healthcare). Band optical density was quantified with the ImageQuantTL software (EG Biosciences).

### Determination of Neuromuscular Junction Innervation

Pups were euthanized with an overdose of Dolethal (20 mg/ml) on PND11. Splenius and longissimus muscles were dissected and fixed for 20 min in 4% PFA at RT. Muscles were quenched for 30 min in 0.1M glycine in PBS and subsequently incubated in Alexa 555-conjugated α-bungarotoxin (α-BTX; 1/250, Thermo Fisher Scientific, B35451) in PBS for 10 min to visualize the post-synaptic endplates. Next, muscles were incubated for 5 min in methanol at −20°C and blocked in 2% BSA diluted in PBS with 0.3% TritonX-100 (blocking solution) for 1 h and subsequently incubated overnight with primary antibodies in blocking solution at 4°C. The following primary antibodies were used to visualize axons and the pre-synaptic terminal, respectively: Alexa 488-conjugated rabbit anti-neurofilament-L C28E10 (1/500, Cell signaling, 2837S) and rabbit anti-synaptophysin YE269 (1/200, Abcam, ab32127). Alexa 647-labeled anti-rabbit antibody (1/500, Life Technologies) was used as a secondary antibody and muscles were incubated for 2 h in blocking solution containing this antibody. Muscles were extensively washed in PBS after each step and all steps were performed at RT unless described otherwise. Image z-stacks were taken at sequential focal planes 1 μm apart for a total depth of ±30 μm with a Leica TSC SP8 confocal laser scanning microscope (Leica Microsystems Heidelberg GmbH) with a HC PL APO CS2 20×/0.75 dry lens. The innervation status of individual post-synaptic endplates was evaluated based on the co-localization of synaptophysin and α-BTX. Fully innervated NMJs were defined by a complete overlap of the endplate with synaptophysin, while partially innervated NMJs were incompletely covered with synaptophysin. Fully denervated NMJs were lacking any presynaptic labeling. Illustrated images are maximum Z-projections created using the ImageJ software by Wayne Rasband (National Institutes of Health). For each smn genotype, EphA4^+/–^ mice were normalized to EphA4^+/+^ mice.

### Determination of Motor Neuron Survival

Pups were euthanized with an overdose of Dolethal (20 mg/ml) on PND11. Lumbar spinal cords were dissected and homogenized in TRIzol (Thermo Fisher Scientific, 15596026) using the MagNaLyser oscillator. Total RNA was precipitated with isopropanol, of which 1 μg was used to prepare cDNA with the SuperScript III First-Strand Synthesis System (Thermo Fisher Scientific, 18080051). Quantitative PCRs were performed with the TaqMAN Fast Universal PCR Master Mix 2X (Thermo Fisher Scientific, 4364103) using 1/10 diluted cDNA and the following Taqman assays (IDT): Chat (Mm01221882_m1), Gapdh (Mm.PT.39a.1) and Polr2a (Mm.PT.58.13811327). PCR reaction was performed in a StepOnePlus instrument (Life Technologies) and relative gene expression was analyzed with the Qbase + software (Biogazelle).

### Statistics

Based on previous results with other modifying treatments in SMNΔ7 mice (Van Meerbeke et al., 2013; Rindt et al., 2015), we estimated a sample size of 13 animals per group to detect a relevant 20% difference in survival with 80% power at an α = 0.05. Similarly, a sample size of 3 animals per group was estimated to detect a relevant 20% difference in fully innervated NMJs with 80% power at an α = 0.05. As researchers were blind for genotype at the time of muscle extraction and analysis, a total of 27 splenius muscles and 25 longissimus muscles were analyzed to ensure a sufficient number of animals in each experimental group.

All statistical analyses were performed with GraphPad Prism software version 7 (GraphPad software Inc). Two-way ANOVA and two-way repeated measures ANOVA with Sidak’s multiple comparison tests were used for multiple group analysis. Due to differences in survival between mice, last weight, righting reflex test and HLS test observations were carried forward to enable repetitive measures statistical analysis.

Survival was analyzed using the Log-rank Mantel–Cox test. ^∗^*p* ≤ 0.05, ^∗∗^*p* ≤ 0.01. All data represents means ± SEM.

## Results

### Loss of EphA4 Does Not Improve Motor Function or Survival in SMNΔ7 Mice

**FIGURE 1:**
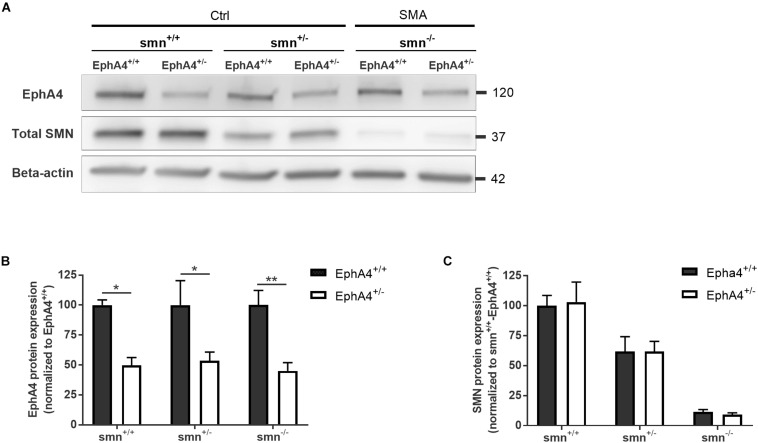
EphA4 levels are reduced in the spinal cord of SMNΔ7 mice, without affecting SMN protein levels. Representative images **(A)** and quantifications **(B,C)** of a Western blot analysis of EphA4 and SMN levels in spinal cord lysates of Ctrl (smn^+/+^ and smn^+/–^) and SMA (smn^–/–^) pups are shown. Beta-actin protein levels were used as a loading control (two-way ANOVA with Sidak’s multiple comparison test, *n* = 4–5 mice/group). ^∗^*p* ≤ 0.05 and ^∗∗^*p* ≤ 0.01. Ctrl, control.

As removal of one EphA4 allele was sufficient to improve the disease phenotype in an ALS mouse model (Van Hoecke et al., 2012), we crossbred EphA4^+/–^ mice with SMNΔ7 mice. SMNΔ7 mice have an average lifespan of approximately 2 weeks and mice show severe motor function abnormalities together with modest loss of motor neurons in the anterior horn of the spinal cord, functional, and morphological abnormalities at the NMJs, muscle denervation, and muscle atrophy (Le et al., 2005; Kariya et al., 2008; Murray et al., 2008; Kong et al., 2009; Lee et al., 2011; Ling et al., 2012). Western blotting of spinal cord lysates at PND8 confirmed a reduction of ±50% of EphA4 protein levels in Ctrl-EphA4^+/–^ (51.7 ± 12.7%) versus Ctrl-EphA4^+/+^ (100 ± 27.2%) mice, and in SMA-EphA4^+/–^ (45.1 ± 13.7%) versus SMA-EphA4^+/+^ (100 ± 24.4%) mice (Figures 1A,B). Reduction of SMN protein was similar in mice with normal versus lower EphA4 expression levels (Figures 1A,C).

**FIGURE 2:**
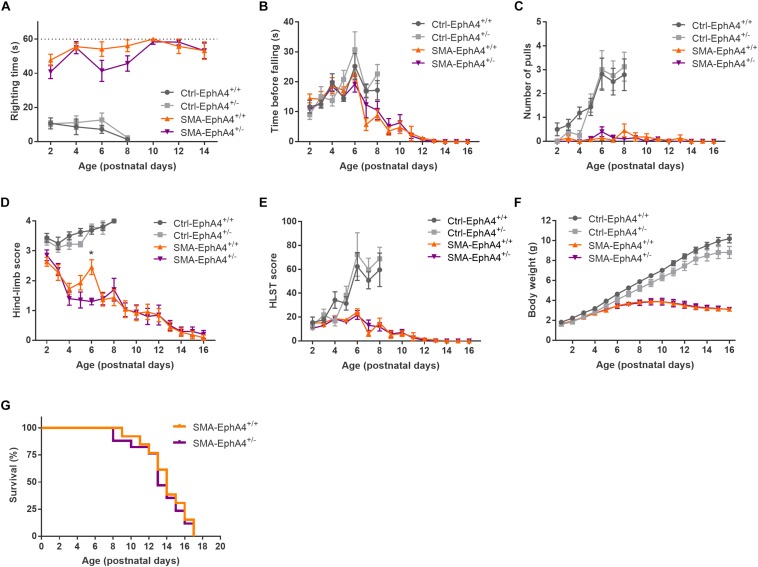
Decrease of EphA4 does not alter motor function and survival in SMNΔ7 mice. Motor performance as assessed in the righting reflex test **(A)** and HLS test **(B–E)**, and body weight **(F)** were monitored during disease progression in SMA-EphA4^+/+^ (*n* = 11) and SMA-EphA4^+/–^ (*n* = 10) pups. Ctrl-EphA4^+/+^ (*n* = 8) and Ctrl-EphA4^+/–^ (*n* = 9) pups were included as controls. Two-way repeated measures ANOVA with Sidak’s multiple comparison test was used to compare Ctrl-EphA4^+/+^ versus Ctrl-EphA4^+/–^ pups and SMA-EphA4^+/+^ versus SMA-EphA4^+/–^ pups. Survival analysis **(G)** in SMA-EphA4^+/+^ (*n* = 13) and SMA-EphA4^+/–^ (*n* = 17) pups (Log-rank Mantel–Cox test). ^∗^*p* ≤ 0.05. Ctrl, control; HLST, hind-limb suspension test.

We evaluated motor function in control and SMA mice with normal versus reduced EphA4 levels with the righting reflex test and the HLS test at regular time points during disease progression. Ctrl-EphA4^+/+^ and Ctrl-EphA4^+/–^ pups showed normal development of motor function, as reflected in the time to right during the righting reflex test (Figure 2A) and in the increased hanging time, number of pulls, and HLST score (Figures 2B–E) during the HLS test. Behavioral analysis of SMA-EphA4^+/+^ pups revealed a compromised righting ability and performance during the HLS test which did not improve by reducing the expression of EphA4 (Figures 2A–E). We monitored weight as an evaluation of general health and muscle mass. While Ctrl-EphA4^+/+^ and Ctrl-EphA4^+/–^ mice showed a continuous gradual increase in body mass, weight gain stagnated in SMA-EphA4^+/+^, and SMA-EphA4^+/–^ mice (Figure 2F) with no difference between the two genotypes. Finally, we investigated whether loss of EphA4 extended lifespan in SMA mice. The average lifespan of SMA-EphA4^+/+^ mice (13.9 ± 2.4 days) was similar to SMA-EphA4^+/–^ mice (13.4 ± 2.7 days) (Figure 2G).

### Decrease of EphA4 Does Not Increase Neuromuscular Junction Innervation in SMNΔ7 Mice

**FIGURE 3:**
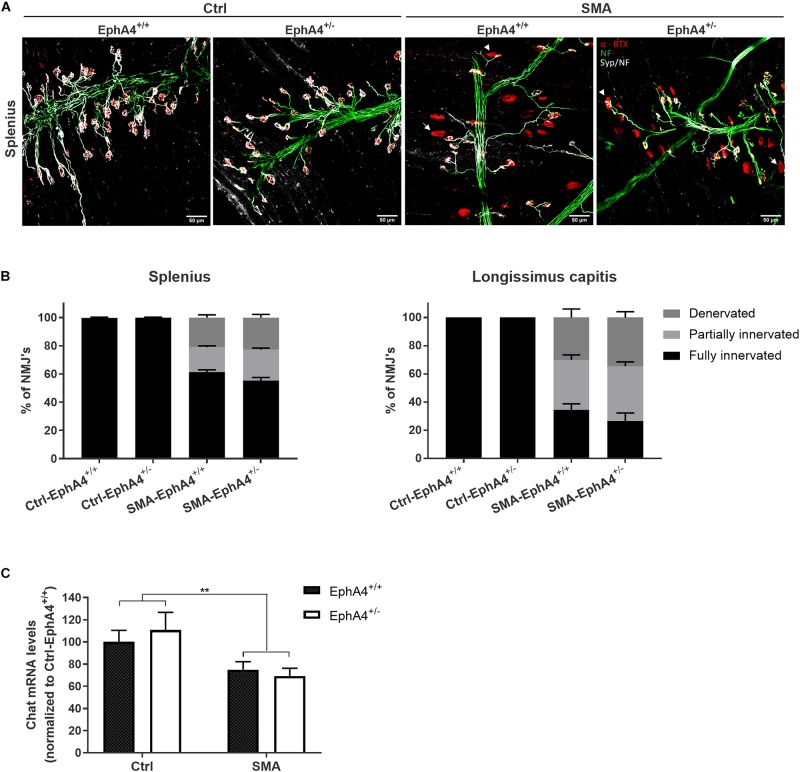
Decrease of EphA4 does not improve neuromuscular junction innervation in SMNΔ7 mice. Innervation status of NMJs in affected muscles was determined in pups at PND11 via immunohistochemical labeling with antibodies specific for NF-L and synaptophysin. Alpha-bungarotoxin was used to label the motor endplates. Representative images **(A)** and quantifications **(B)** of fully innervated, partially innervated (arrowhead) and denervated (arrow) NMJs in the splenius and longissimus capitis muscles of Ctrl-EphA4^+/+^ (*n* = 6), Ctrl-EphA4^+/–^ (*n* = 4), SMA-EphA4^+/+^ (*n* = 9–10) and SMA-EphA4^+/–^ (*n* = 6–7) pups are shown (two-way ANOVA with Sidak’s multiple comparison test). **(C)** Quantification of a quantitative PCR analysis of Chat mRNA expression levels in the lumbar spinal cord of Ctrl-EphA4^+/+^ (*n* = 5), Ctrl-EphA4^+/–^ (*n* = 4), SMA-EphA4^+/+^ (*n* = 8) and SMA-EphA4^+/–^ (*n* = 7) pups at PND11 (two-way ANOVA with Sidak’s multiple comparison test). Expression data was normalized to Gapdh and Polr2a. ^∗∗^*p* ≤ 0.01. Scale bar = 50 μm. NF-L, neurofilament-L; Ctrl, control.

Since lowering EphA4 did not improve motor function, nor survival in SMNΔ7 mice, we confirmed the lack of a modifying role for EphA4 by evaluating innervation status of NMJs of two severely affected axial muscles in the SMNΔ7 mouse model, the splenius and longissimus capitis muscles (Van Meerbeke et al., 2013). At PND11, all NMJs were fully innervated in Ctrl-EphA4^+/+^ and Ctrl-EphA4^+/–^ mice in both muscles (Figures 3A,B). In contrast, a profound denervation of NMJs occurred in SMA-EphA4^+/+^ mice, with only 61% and 34% of NMJs remaining fully innervated in the splenius and longissimus muscles, respectively (Figures 3A,B). Loss of EphA4 did not affect the innervation status of the NMJs in both muscle types (Figures 3A,B). As SMNΔ7 mice also present with a modest loss of motor neurons in the spinal cord (Le et al., 2005), we additionally evaluated expression levels of the motor neuron marker gene Chat in the lumbar spinal cord of Ctrl and SMA mice at PND11. As expected, we observed a reduction of Chat mRNA levels in SMA-EphA4^+/+^ mice in comparison to Ctrl mice. Loss of EphA4 in SMA mice (SMA-EphA4^+/–^) did not alter Chat mRNA levels, suggesting no effect of reducing EphA4 expression on motor neuron survival in SMNΔ7 mice (Figure 3C).

## Discussion

Previously, we identified EphA4 as a disease modifier of ALS in both zebrafish and rodent models for this disease (Van Hoecke et al., 2012). Knockdown of EphA4 also rescued the axonal outgrowth deficits in a zebrafish model for SMA (Van Hoecke et al., 2012). In this study we aimed to validate the disease modifying potential of reduced EphA4 protein levels in the SMNΔ7 mouse model for severe SMA by evaluating NMJ innervation, motor neuron loss, motor function and survival. Loss of one EphA4 allele in this mouse model did not enhance any of these parameters despite clear reduction of EphA4 protein levels.

Both in ALS and SMA patients as well as in mouse models, motor neurons are degenerating via a “dying-back mechanism” in which pathology starts at the NMJ and progresses toward the cell body along the axon (Frey et al., 2000; Fischer et al., 2004; Murray et al., 2008; Bowerman et al., 2012). However, our findings in the SMNΔ7 mouse model are in contrast with previous results in the mutant SOD1^G93A^ mouse model for ALS in which similar reduction of EphA4 levels ameliorated motor neuron disease progression (Van Hoecke et al., 2012). Clinical and pathological dissimilarities between these diseases and mouse models could explain the differential effect of EphA4 modulation on disease outcome.

First, disease severity varies significantly in ALS versus SMA. ALS is an adult onset disease resulting in death 3–5 years after symptom onset. In contrast, clinical onset in severe SMA patients starts shortly after birth and rapidly progresses to death before the age of 2 years (Lunn and Wang, 2008; Brown and Al-Chalabi, 2017). In mouse models, this variation is reflected in the NMJ pathology where first signs are observed during adult stages in the mutant SOD1^G93A^ ALS mouse model, while this already happens in the neonatal phase in the SMNΔ7 mouse (Frey et al., 2000; Pun et al., 2002; Kariya et al., 2008; Murray et al., 2008; Kong et al., 2009; Ling et al., 2012). Moreover, disease progression is much faster in the SMNΔ7 mouse model and results in death within 2 weeks, while mutant SOD1^G93A^ mice survive for a few months following disease onset (Gurney et al., 1994; Le et al., 2005). Therefore, the period during which compensatory re-innervation of NMJs could occur, could have been too short and therefore insufficient to modify SMA pathology and phenotype.

Second, compensatory sprouting and re-innervation is limited in SMA patients and mouse models for severe SMA, while these mechanisms are well-established phenomena in ALS patients and in the mutant SOD1^G93A^ mouse model (Hansen and Ballantyne, 1978; Bromberg et al., 1993; McComas et al., 1993; Frey et al., 2000; Cifuentes-Diaz et al., 2002; Pun et al., 2002; Bosch-Marcé et al., 2011). Pre-synaptic neurofilament accumulations and instability of the NMJs due to immaturity and decreased numbers of terminal Schwann cells, contribute to the observed sprouting and re-innervation deficiencies in severe SMA (Kariya et al., 2008; Murray et al., 2008, 2013; Kong et al., 2009; Lee et al., 2011). Hence, even if the axonal intrinsic sprouting capacity would increase via EphA4 modulation in severe SMA mice, this might not be sufficient to restore NMJ innervation, as the NMJs are severely affected.

Third, in ALS patients and mouse models different types of motor neurons exhibit diverse patterns of vulnerability, which is determined by EphA4 expression levels. Spinal motor neurons with the highest EphA4 levels (fast-twitch fast fatigable, FF) are most vulnerable and degenerate first, while motor neurons with lower EphA4 levels (slow, S) are more resistant (Frey et al., 2000; Pun et al., 2006; Van Hoecke et al., 2012). In SMNΔ7 mice, no clear correlation between motor neuron type and vulnerability exists, as muscles innervated by both FF and S motor neurons can be equally affected (Ling et al., 2012). Therefore, EphA4 might not contribute to motor neuron vulnerability in SMA, limiting its therapeutic potential in this disease.

Further research will need to clarify whether loss of EphA4 might be beneficial in mouse models representing milder forms of the disease with a broader therapeutic window for intervention, such as Smn^+/–^ and SMN A2G transgenic mice. In addition to a slower disease progression, sprouting events have been reported in these models (Monani et al., 2003; Simon et al., 2010), possibly enabling a greater potential for beneficial effects from reduced EphA4 levels as well. Moreover, it might be interesting to investigate the potential of reducing EphA4 levels in combination with other therapeutic disease-ameliorating strategies. Encouraging data come from two studies in which administration of Plastin-3 (PLS3) or suppression of neurocalcin delta (NCALD) failed to modify the severe SMA phenotype, while combining these strategies with an SMN protein increasing compound had additional benefit (Kaifer et al., 2017; Riessland et al., 2017). Therefore, a similar strategy might be useful for EphA4 knockdown in the SMNΔ7 mouse model.

The present study was limited by the partial 50% reduction of EphA4. Although removal of one EphA4 allele (EphA4^+/–^) was sufficient to improve the disease phenotype in an ALS mouse model (Van Hoecke et al., 2012), this could be insufficient to modify the severe SMA phenotype and a more dramatic reduction of EphA4 might be required. However, this is unfeasible to investigate as EphA4^–/–^ mice show very low birth rates and develop a “hopping gait” phenotype, limiting the chance of obtaining SMA-EphA4^–/–^ mice, and the assessment of motor function (Dottori et al., 1998; Kullander et al., 2003; Van Hoecke et al., 2012).

In conclusion, in contrast to previous positive results in a zebrafish model for SMA and in the mutant SOD1^G93A^ mouse model for ALS, our work demonstrates that loss of one EphA4 allele is not sufficient to improve the innervation of the NMJs, motor neuron survival, motor function and survival in the SMNΔ7 mouse model for severe SMA. We hypothesize that this is due to either a too severe clinical phenotype in the SMNΔ7 mouse model with a too short time window for possible re-innervation, or to a limited therapeutic potential of reduced EphA4 in SMA.

## Data Availability Statement

The datasets generated for this study are available on request to the corresponding author.

## Ethics Statement

The animal study was reviewed and approved by the Ethical Committee for Animal Research of the University of Leuven, Leuven, Belgium.

## Author Contributions

LP performed and coordinated all the experiments, analyzed the data, and wrote the manuscript. SS provided the additional support for the collection and staining of muscles, and for the quantification of neuromuscular junction innervation. MT, AL, AS, LS, and AdB provided the technical assistance during some experiments. SS, LR, LVDB, PVD, and WR supervised the project. RL supervised and wrote the manuscript. All authors contributed to the final manuscript.

## Conflict of Interest

The authors declare that the research was conducted in the absence of any commercial or financial relationships that could be construed as a potential conflict of interest.

## Abbreviations

HLS: hind-limb suspension
PND: postnatal day.
